# Ambient temperatures associated with reduced cognitive function in older adults in China

**DOI:** 10.1038/s41598-023-44776-2

**Published:** 2023-10-13

**Authors:** Kun Hou, Xia Xu

**Affiliations:** 1 School of Remote Sensing and Geomatics Engineering, Nanjing University of Information Science and Technology, Nanjing, 210044 China; 2 Jiangsu Province Hydrology and Water Resources Investigation Bureau, Nanjing, 210029 China

**Keywords:** Environmental sciences, Risk factors

## Abstract

The cognitive function status of older adults determines the social function and living quality of older adults, which is related to the healthy development and stability of the society. However, the impact of high or low ambient temperature on cognitive function in older adults remains unclear. Based on data from the Chinese Longitudinal Healthy Longevity Survey (CLHLS), we comprehensively assessed the impact of ambient temperature on the cognitive function of older adults in this study. The findings exhibited that for each 1 °C ascent in monthly temperature of high temperature, the examination score of global cognitive function of older adults decreased by 0.48 (95% CI 0.21–0.74), which was greater than that of 0.14 (95% CI 0.06–0.25) for each 1 °C reduction in low temperature. Overall, the detrimental effect of high temperature on cognitive function in older adults was more significant than that of low temperature, including on the five sub-cognitive functions involved. Our research provides vital technical guidance and reference for the health protection and prevention of cognitive function of older adults in specific external environmental conditions under the current climatic variation and temperature rise.

## Introduction

The global climate is changing at an unpredictable rate^1^. Climate change is already approaching the 1.5 °C threshold, and even with substantial reductions in the emissions of greenhouse gases, the magnitude of climate change cannot be contained below the threshold of 2 °C, resulting in a global temperature rise of 2 °C by 2050^2,3^. Non-optimal temperature exposure constitutes a growing threat to public health and outcomes, and affects multiple health outcomes directly or indirectly through different physiological mechanisms simultaneously^4,5^, ranging from increased risk of gestational hypertension or preterm birth^6,7^, ascended mortality from respiratory and circulatory disease and stroke^8–11^, significantly reduced the health level of workforce^12,13^, enhanced visits of the emergency department^14^, and the negative effects on cognitive function in older adults^15,16^.

Cognitive decline in older adults is mainly manifested as perception disorders, memory impairment or disturbance of thought, prone to dull reactions, memory loss or defects, and accompanied by a decline in daily living ability, which further develops into depression, Alzheimer's disease, Parkinson's disease, and dementia^17–20^, seriously affecting the social function and quality of life of older adults^21^. Compared with the optimal temperature, the average scores of cognitive function of the examined older adults in extreme cold and heat both decreased, and episodic memory was more sensitive to heat exposure, while semantic memory and executive function were the two cognitive domains that were sensitive to cold exposure in Germany^16^. Older adults with the risk of low MMSE scores demonstrated a significant U-shaped association with residential ambient temperature and culminated at extreme high or low temperatures^15^. The risk of mild cognitive impairment among community-dwelling older adults in Taiwan aggrandized with rising ambient temperature^22^. Lower temperatures were associated with an increased risk of impaired cognitive ability^23^ and lower cognitive scores in older adults^24^. Fine particulate matter of air pollutants such as PM_2.5_ has been widely confirmed to be associated with cognitive decline in older adults^20,25,26^. Precipitation has also been found to indirectly affect cognitive function in older adults by intervening in the concentrations of particulate matter in the atmosphere^27,28^. In addition, excessive rain would force the older adult to spend more time at home, reduce their interaction and communication, and enhance the sense of isolation, which is not conducive to the development of cognitive functions^29,30^. These findings provide a plausible hypothesis for the linkage between temperature variations and cognitive decline in older adults and the research directionality on how to control for these confounding factors to highlight the effect of temperature on cognitive function in older adults.

Several studies had investigated and revealed the heterogeneity of the impacts of environmental temperature exposure on cognitive function in terms of different subgroups of older adults, in which the quantitative relationship between older adults with different sociodemographic characteristics, geographical distribution^15,31^, individual physiological diatheses^16,22,23^, and lifestyles or habits^15,23^ and temperature variations were analyzed separately. Some of these heterogeneity results were significant, which verified the necessity of exploring the differences in the cognitive function of the Chinese older adults influenced by temperature.

Previous studies have paid close attention to the short-term effects of temperature (i.e., the scale of daily temperature) on the cognitive function of older adults, with less involvement of longer-term temperature (i.e., the scale of monthly temperature)^15,16,23,24^. The variations of monthly temperature have been identified as exerting a negative effect on various health outcomes, including mortality and suicide, and the changes and patterns of such effects differ from those of daily temperature^32,33^. The impact of longer-term monthly temperature variations on cognitive function in older adults remains unclear. Additionally, there are few studies on the relationship between ambient temperature and the cognitive function of older adults in China, where the effects of high and low temperature exposure need to be further elucidated.

In this study, based on the cognitive function examination data of the Chinese Longitudinal Healthy Longevity Survey (CLHLS), combined with high-precision temperature data, the effect of temperature variations on the decline of cognitive function of older adults in China was explored, where the overall score of cognitive function and five types of sub-cognitive functions including the general ability of cognitive function, memory ability, attention and calculation ability, reaction ability, and language comprehension and self-coordination ability were assessed separately. We used a series of refined Bayesian spatiotemporal geo-statistical models which fully accounted for space–time variations and revealed the complex nonlinear associations between temperature variations and the health outcomes for sizeable geographic scale studies ^33^. The displacement effect of temperature combined with the distributed lag effect was investigated, whose overall lag effect was referred to as a reflection of the overall effect of temperature on the examination score of cognitive function. Subsequently, we estimated the heterogeneity of the effects of temperature variations on the cognitive function of older adults with respect to different socioeconomic demographic characteristics. Our study contributes to a comprehensive understanding of the unequal influence of temperature exposure on cognitive decline in different subgroups of older adults in China, and provides key technical guidance for the health protection of cognitive function in older adults under specific external environment.

## Materials and methods

### Cognitive function examination data

**Figure 1:**
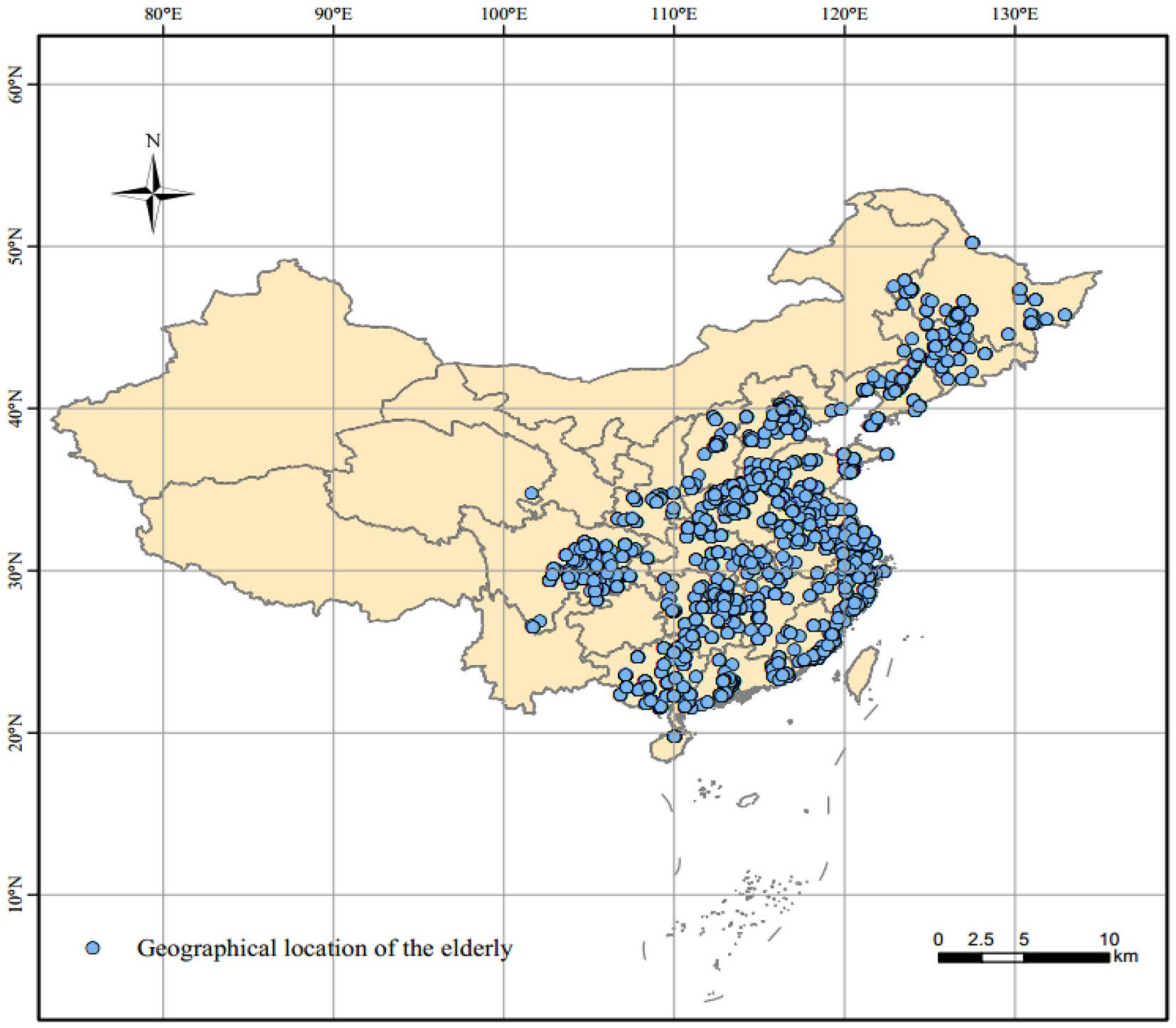
Geographic spatial distribution of older adults.

The cognitive function data of older adults were derived from the Chinese Longitudinal Healthy Longevity Survey (CLHLS). We used three waves of survey data related to the older adults from the CLHLS in our study, including 2008–2014, 2011–2014, and 2014–2018 (https://opendata.pku.edu.cn/). The waves of 2008–2014, 2011–2014, and 2011–2014 included 16,954, 9,765, and 7,192 older adults, respectively. We eliminated the data with missing information on the older adults (including geographical location, socioeconomic attributes, age, gender, and ethnicity), incomplete cognitive function examinations (less than five sub-cognitive functions), and duplicate IDs of the older adults. Eventually, a total of 17,791 older adults were valid in the study. The contents of the survey included individual micro-data such as cognitive function, social family demographic characteristics, living habits, economic status, family structure, and concomitant morbidity of older adults. Figure 1 illustrates the geographical distribution of older adults, of which 718 different districts and counties in mainland China are covered.

The Mini Mental Status Examination (MMSE) is widely utilized as a universal measure of cognitive function^15^, and is used to examination and score the cognitive function for each older adult (i.e., MMSE-score). The examination covers five major aspects, including the general ability of cognitive function, memory ability, attention and calculation ability, reaction ability, and language comprehension and self-coordination ability of older adults, involving a total of 24 examination items. Each examination item was scored according to whether the answer was correct, with each older adult receiving a global score ranging from 0 to 30, where higher scores represented greater cognitive function for older adults. The MMSE-scores of the five aspects included in the cognitive examination were calculated separately in the study for exploring the effect of temperature on the sub-cognitive functions simultaneously.

### Meteorological data

We obtained meteorological data from the 1-km monthly mean temperature and precipitation dataset for China (https://data.tpdc.ac.cn/en/data/)^34^, where monthly mean temperature and precipitation covering the entire time span of the study were available. The dataset was spatially downscaled from the Climatic Research Unit (CRU) time series dataset with the climatology dataset using delta spatial downscaling^35^, and was evaluated using observations collected in 1951–2016 by 496 weather stations across China^36^. We used the grid to derive the monthly average temperature and precipitation data for each older adult. Rain may discourage older adults from leaving their homes with potential consequences for social isolation, decreased physical activity, and cognitive decline^29,30,37^. At the same time, precipitation affects the concentrations of fine particulate matter (e.g., PM_2.5_) in the atmosphere^27,28^. The concentrations of particulate matter have been widely confirmed to be associated with cognitive decline^25,26^, whereby linking precipitation with cognitive decline. Therefore, we controlled for precipitation as the interference factor in the model.

### PM_2.5_ concentrations data

Air pollution has been confirmed to exert a certain impact on cognitive function in older adults^20,25,26^, thus we refer to the pollutant of PM_2.5_ to control for and reflect the interference effect of air pollution on the cognitive function of older adults in the study. PM_2.5_ data were derived from the National Earth System Science Data Center, National Science and Technology Infrastructure of China (http://www.geodata.cn), where seamless near-surface PM_2.5_ concentration data is available across the Chinese mainland. The temporal and spatial resolutions of this dataset were daily and 1 km, respectively, and we obtained daily average PM_2.5_ concentration data over the entire time span of the research area, which was utilized to geocode and obtain the monthly average PM_2.5_ concentrations for each district and county.

### Methods

#### Study design

We conducted a spatiotemporal series analysis with secular trend and geographical distribution, in which the cognitive function examination scores of older adults in various districts and counties were appropriately linked to the corresponding monthly mean temperature. The time span of this study is from 2008 to 2018, with a wide range of monthly average temperatures of − 13.3 to 30.8 °C, and the spectrum of older adults involved covers 23 provinces, municipalities and autonomous regions and 85% of the entire population in China. The wide range of temperatures and diverse demographic characteristics of older adults afford a natural condition of experiment for thoroughly exploring and identifying the effects of environmental temperature exposure on cognitive function and its heterogeneity in older adults.

#### Investigation of the correlation for environmental temperature and cognitive function of older adults

Precipitation and PM_2.5_ were referred to as the interference factors of the external environment in assessing the association between ambient temperature and cognitive function. The cognitive function examination score of each older adult was correlated with the monthly average temperature, precipitation, and PM_2.5_ concentrations of the district or county where older adults located, reflecting the exposure of environmental factors of older adults in that month. Both the meteorological variables of temperature and precipitation and the air pollutant of PM_2.5_ span from January 2008 to December 2018.

We computed the mean score of cognitive function of older adults in accordance with the quartiles of average monthly temperature, and ascertained that older adults exposed to low and high temperatures were associated with lower cognitive function scores relative to the reference temperatures. The reference temperature was defined as at which the cognitive function scores were highest (see below). Subsequently, we leveraged a series of sophisticated geographic spatiotemporal statistical models to explore the association between the variation of ambient temperature and the cognitive function of older adults, in which the environmental factors including precipitation, PM_2.5_ concentrations, and the respective effects of time and space and the interaction effect of time–space were controlled.

#### Generalized linear additive model (GLAM)

We denote the MMSE-score for each older adult in month *t* of county *c* as *y*_*ic*_, where the maximum values for *c* and *t* are 718 and 12, corresponding to the 718 administrative units involved in this study and the 12 months of the year, respectively. *y*_*ic*_ is a continuous variable, reflecting that the MMSE-scores of all older adults varies from 0 to 30. We conduct and derive a generalized linear additive model, as shown in Eq. (1):

1 $$\eta_{i} { = }g\left( {\mu_{i} } \right) = \alpha + \sum\limits_{j = 1}^{J} {\beta_{j} } z_{ji} + \sum\limits_{k = 1}^{K} {f_{k} (} u_{ki} )$$

where *α* is the intercept, *z*_*j*_ represents the *j*th covariate and apply a linear relationship with $\eta_{i}$, and *β*_*j*_ is the regression coefficient of the *j*th covariate. *μ*_*i*_ = *E(y*_*ic*_*)* denotes the expectation of MMSE-score and follows a Gaussian distribution. *g* indicates the identity link function. *u*_*k*_ accounts for the influence term with $\eta_{i}$, and *f* represents the corresponding functional relationship.

#### Bayesian spatiotemporal model

We utilized monthly average temperature as the exposure index (°C). Based on the GLAM, we explored the relationship of cognitive function with environmental temperature using a Bayesian spatiotemporal model^33^, leveraging variations over time and space to deduce the associations between ambient temperature and the MMSE-score, which is shown in Eq. (2):

2 $$\begin{aligned} {\text{Score}}_{{\text{county-time }}} & = \left( {\alpha_{0} + \beta_{0} \times {\text{ time }}} \right){ + }f{\text{ temperature}}_{\text{county-time}} \\ & \quad + \left( {\alpha_{{\text{county }}} + \beta_{{\text{county }}} \times {\text{ time }}} \right) + \left( {\alpha_{{\text{month }}} + \beta_{{\text{month }}} \times {\text{ time }}} \right) \\ & \quad + \left( {\zeta_{{\text{county-month }}} + \psi_{{\text{county-month }}} \times {\text{ time }}} \right) + \nu_{{\text{time }}} + \varepsilon_{{\text{county-time }}} + \beta 1preci + \beta 2PM_{2.5} \\ \end{aligned}$$

where Score_county-time_ denotes the MMSE-score of each older adult in month *t* of county *c*. temperature_county-time_ represents the monthly average temperature in county *c*. *f* indicates the temperature response function, including the models of higher-order polynomials and splines. *α*_*0*_ indicates the common intercept, and *β*_*0*_ represents the general slope with overall time. *α*_*county*_, *β*_*county*_ represent the intercept of specified-county and the corresponding specified-county slope, which are interpreted as spatially structured versus unstructured random effects between different neighboring counties and the variation of such random effects with overall time, respectively. These county-level random intercepts and slopes are modeled by using the Besag, York and Mollie spatial model^38^, which fully identifies the spatial structured effects with intrinsic conditional autoregressive priors and captures the independent identically distributed Gaussian random spatial unstructured effects^33^. *α*_*month*_, *β*_*month*_ indicate the intercept of specified-month and the corresponding specified-month slope, which account for the differential random effects between adjacent months and the variation with overall time, respectively. The first-order random walk prior was used to simulate the monthly random intercept and slope, which was confirmed to be effective in delineating the smoothly varying trends^33^. December is set adjacent to January because of the cyclic structure of the random walk^39^. *ζ*_county-month_, *ψ*_county-month_ represent the intercepts and slopes of the county-month interactions, accounting for the changes in the MMSE-score levels and trends in a particular county across various months, respectively. We use a first-order random walk to capture the nonlinear variation along the entire time scale (in months), ν_time_^39^. *ε*_*county-time*_ denotes an over-dispersion term, which is interpreted as the potential interference effect not involved by other terms in the model, simulated as *N(0, σ*^*2*^_*ε*_*)*^40^. *preci* and *PM*_*2.5*_ denote precipitation and the air pollutant of PM_2.5_, reflecting the interference effects of precipitation and air pollution, respectively. *β*_*1*_ and *β*_*2*_ represent the corresponding coefficients, respectively. The meanings of the remaining terms remain unchanged as in Eq. (1).

We assessed the association between the variation of temperature and cognitive function of older adults in two steps. First, we derived the preliminary nonlinear relationship between temperature and the MMSE-score by fitting different functions of *f* including polynomial, b-spline, natural cubic spline with 3 nodes, and natural cubic spline with 5 nodes. We multiplied the temperature coefficients obtained using the fitting functions *f* by the temperature values over the entire range, which were used to derive the contribution to the deviation in the score for a specific temperature. Next, we set a reference temperature, whose specific contribution is used as a reference value to calculate how much the score contribution of other temperatures departs from it. This is considered as the change in the MMSE-score^32^, which reflects the relationship between different temperatures and the score of cognitive function. The selection of the reference temperature does not interfere with the assessment of the linkage between temperature and the MMSE-score. Here we chose -7 °C as the reference temperature to facilitate the interpretation of the results, which suggests that the impacts of low and high temperatures were placed on either side of the reference temperature. The Deviance Information Criterion (DIC)^41^ is used to choose the optimum fit from the four fitting functions, of which the corresponding DIC value exhibits the minimum. The examination score of cognitive function included five aspects of the general ability of cognitive function, memory ability, attention and calculation ability, reaction ability, and language comprehension and self-coordination ability of older adults. Based on the optimal fitting function selected by model (2), we assessed the relationship between these five different types of sub-cognitive function and temperature, respectively.

Subsequently, we divided the temperature range into different intervals according to the relation curve between temperature and the change of MMSE-score, in which the linear effect of temperature increase or decrease on MMSE-score was individually quantified, and the model was shown in Eq. (3):

3 $$\begin{aligned} {\text{Score}}_{{\text{county-time }}} & = \left( {\alpha_{0} + \beta_{0} \times {\text{ time }}} \right){ + }\beta {\text{ temperature}}_{\text{county-time}} \\ & \quad + \left( {\alpha_{{\text{county }}} + \beta_{{\text{county }}} \times {\text{ time }}} \right) + \left( {\alpha_{{\text{month }}} + \beta_{{\text{month }}} \times {\text{ time }}} \right) \\ & \quad + \left( {\zeta_{{\text{county-month }}} + \psi_{{\text{county-month }}} \times {\text{ time }}} \right) + \nu_{{\text{time }}} \\ & \quad + \varepsilon_{{\text{county-time }}} + \beta 1preci + \beta 2PM_{2.5} \\ \end{aligned}$$

where *β* represents the coefficient of the temperature variable, which is interpreted as the change of MMSE-score for every 1 °C rise or decrease in monthly mean temperature^33,42^.

#### Displacement effect of temperature

The displacement effect of temperature refers to the lag and lead effect of temperature, which represents the influence of temperature in the previous months and the following months on the MMSE-score of older adults, respectively^32^. Combined with the distributed lag effect of temperature^43^, we further construct the following Bayesian spatiotemporal model to compute the displacement effect of temperature, as shown in Eq. (5):

4 $$\begin{aligned} {\text{Score}}_{{\text{county - time }}} & = \left( {\alpha_{0} + \beta_{0} \times {\text{ time }}} \right){ + }\left( {\sum\limits_{{L{ = }0}}^{l} {\beta^{L} {\text{temperature}}_{{{\text{(county - time) - }}L}} } } \right) \\ & \quad + \left( {\alpha_{{\text{county }}} + \beta_{{\text{county }}} \times {\text{ time }}} \right) + \left( {\alpha_{{\text{month }}} + \beta_{{\text{month }}} \times {\text{ time }}} \right) \\ & \quad + \left( {\zeta_{{\text{county - month }}} + \psi_{{\text{county - month }}} \times {\text{ time }}} \right) + \nu_{{\text{time }}} + \varepsilon_{{\text{county - time }}} + \beta 1preci + \beta 2PM_{2.5} \\ \end{aligned}$$

in which *β*_L=0_ accounts for the impact of the temperature of the current month, and *β*_L=1_ denotes the impact of the temperature of the previous month. Here, the value of *l* is set to 3, which accounts for the maximum length of the lag effect of temperature to be 3 months before the current month^32^. The sum of the coefficients of *β*_*L*=*0*_ + *β*_*L*=*1*_ + *β*_*L*=*2*_ + *β*_*L*=*3*_ represents the overall lag effect of temperature on MMSE-score^32^. The same applies to the lead effect, which refers to the impact of next month's temperature on the MMSE-score of older adults. The maximum length of the lead effect of temperature is also set to 3 months. Subsequently, we calculate the respective displacement effect for low and high temperature intervals.

We utilized the Integrated Nested Laplace Approximation (INLA)^33^ based on Bayesian statistical theory to infer and calculate the effect of temperature on the MMSE-score of older adults. INLA is a deterministic approximate Bayesian inference method which returns accurate parameter estimates in less time and is an effective alternative to Markov Chain Monte Carlo (MCMC)^42,44^.

#### Heterogeneity analysis

On the basis of the impacts of ambient temperature exposure on the cognitive function in older adults, we assessed the heterogeneity of MMSE-score affected by low and high temperature exposure by grouping older adults according to the socioeconomic demographic characteristics of gender, age, residential area, educational level, total household income, and ethnicity. We used Eq. (4) to estimate the overall effect of low or high temperature on the MMSE-score of global cognitive function in each subgroup. Equation (5) was utilized to estimate the significance of differences for the temperature effects across various subgroups.

5 $$\left( {\hat{Q}_{1} - \hat{Q}_{2} } \right) \pm 1.96\sqrt {S\hat{E}_{1} + S\hat{E}_{2} }$$

where Q_1_ and Q_2_ indicate the temperature effects between any two subgroups, whose standard deviations are represented by SE_1_ and SE_2_. As in previous studies^5,45,46^, we ascertained the alteration of temperature effect by an indicator of ≥ 2 to be significant and noteworthy, which suggested that the corresponding grouping criteria were the determinant factors affecting the MMSE-score in older adults.

### Sensitivity analysis

We varied the combination of confounding factors in model (3), including the variables of county-specific intercept and corresponding time-slope α_county_ and β_county_, the month-specific intercept and slope over time α_month_ and β_month_, the county-month interaction intercept over time and slope term α_county-month_, β_county-month_, precipitation and PM_2.5_ to detect the sensitivity and robustness of the Bayesian spatiotemporal model used for the results of the study. We used the adjusted model to estimate the overall effect of high and low temperature on the global cognitive function in older adults, and the results were shown in Supplementary Table 1, which confirmed the reliability of our results.

### Ethical approval

The Ethical Committee of Peking University gave its approval before the study began (Registration Number IRB00001052-13074).

## Results

### Relationship between environmental temperature and the MMSE-score of cognitive function

We used three waves of the cognitive function examination data related to the older adults from the CLHLS in our study, including 2008–2014, 2011–2014 and 2014–2018. We eliminated the data with missing information on the older adults (including geographical location, socioeconomic attributes, age, gender, and ethnicity), incomplete cognitive function examinations (less than five sub-cognitive functions), and duplicate IDs of the older adults. Eventually, a total of 17,791 older adults were valid in the study. The examination for cognitive function in older adults included five sub-functions of general ability, memory ability, attention and calculation ability, reaction ability, and language comprehension and self-coordination ability, whose quantitative associations with temperature variations were assessed separately in the study (see below).

We utilized the Bayesian spatiotemporal model to explore the association between ambient temperatures and cognitive function of older adults, where different fitting functions including 3 knots of natural cubic spline, 5 knots of natural cubic spline, polynomial, and B-spline were used to simulate and characterize the effect of temperature variations on the MMSE-score of global cognitive function (see Methods). The reference temperature was preset, whose effect on the MMSE-score was considered as a reference to calculate the relative change with respect to the impact of other temperatures on the cognitive function^10^. Here, we chose − 7 °C as the reference temperature for facilitating the interpretation of the results (see Methods). The Deviance Information Criterion (DIC) was utilized to choose the optimal fitting function of polynomial, where the corresponding DIC value approached the minimum^46^. Then we used the optimal fitting function of polynomial to assess the association between environmental temperature and five various types of sub-cognitive functions of older adults, respectively.

**Figure 2:**
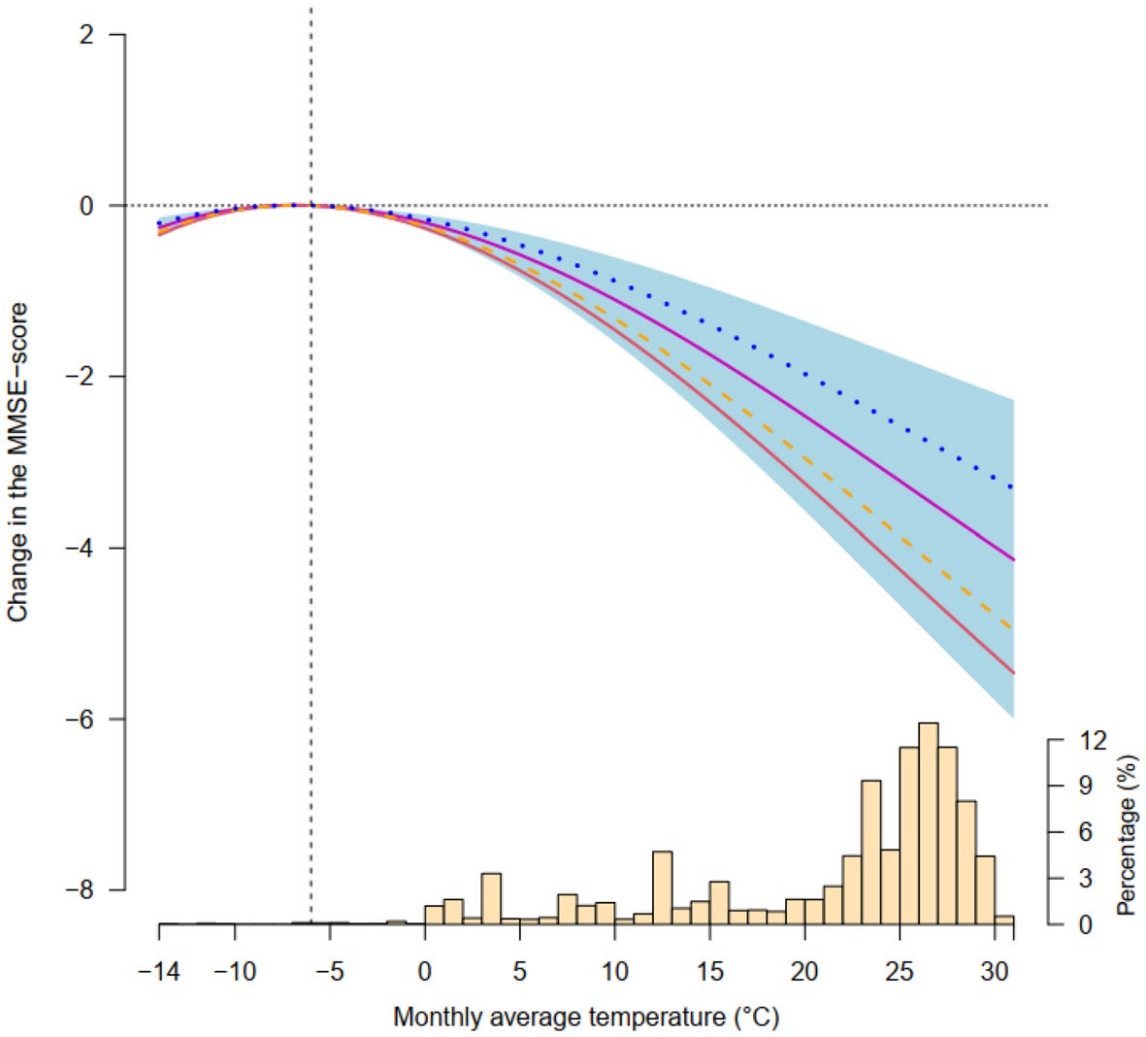
Relation curve between temperature variation and the MMSE-score of global cognitive function. The red, purple, blue and orange dashed lines represent the fitting functions of B-spline, polynomial, natural cubic spline of 3 nodes and natural cubic spline of 5 nodes, respectively. The light blue shading represents the 95% confidence interval of the polynomial function fitting curve, and the histogram on the abscissa axis indicates the distribution of monthly mean temperature.

There was an inverse U-shaped association between monthly mean temperature and the MMSE-score of global cognitive function (Fig. 2). The MMSE-score at extreme cold (− 14 °C) and hot temperatures (31 °C) declined by − 0.34 (95%CI − 0.42 to 0.26) and − 4.23 (95%CI − 4.45 to 2.46), respectively, in comparison to the reference temperature. We observed that the MMSE scores are superior when temperature is approximately around − 7 °C, and are expected to decrease when temperature departs from it. The MMSE-score of the global cognitive function decreased as the temperature increased above − 7 °C, and as the temperature approached the highest, the cognitive function score declined substantially. Low temperatures below the reference temperature were also found to be associated with a decrease in the MMSE-score.

**Figure 3:**
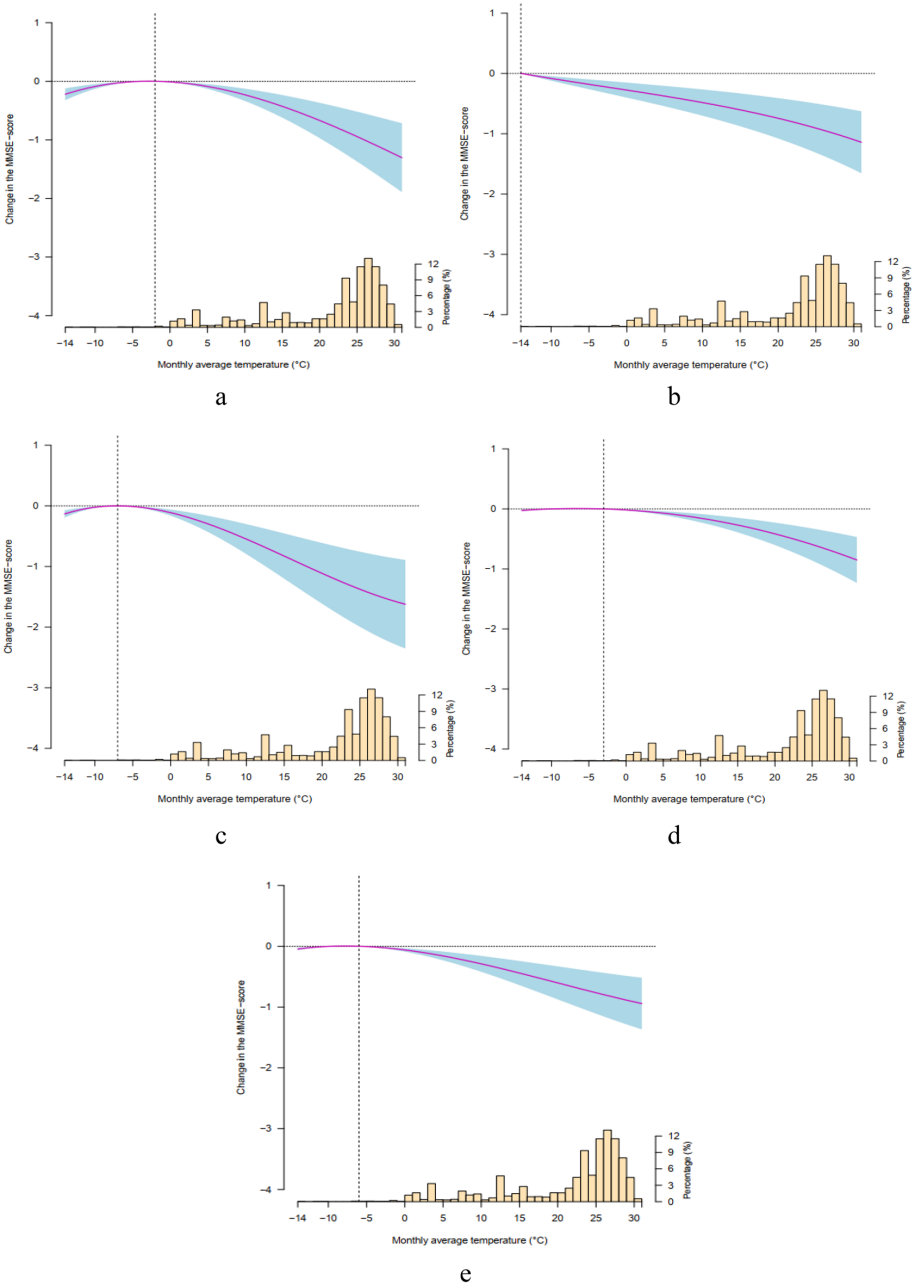
Relation curves between temperature variation and the MMSE-score of various sub-cognitive functions. (**a**) General ability. (**b**) Reaction ability. (**c**) Attention and calculation ability. (**d**) Memory ability. **e**, language comprehension and self-coordination ability. The purple line represents the fitting function of polynomial, the light blue shading represents the 95% confidence interval of the polynomial function fitting curve, and the histogram on the abscissa axis indicates the distribution of monthly mean temperature.

Figure 3 illustrates the change curves of the relationship between ambient temperatures and different sub-cognitive functions of older adults, where both the temperatures below and above the reference value accelerate the decrease of MMSE-score except for the effects of low temperatures on the reaction ability, memory ability, and language comprehension and self-coordination ability. The adverse effects of high temperature on the sub-cognitive functions of older adults were more prominent than that of low temperature.

### Quantitative effects of monthly temperature variations on cognitive function

**Figure 4:**
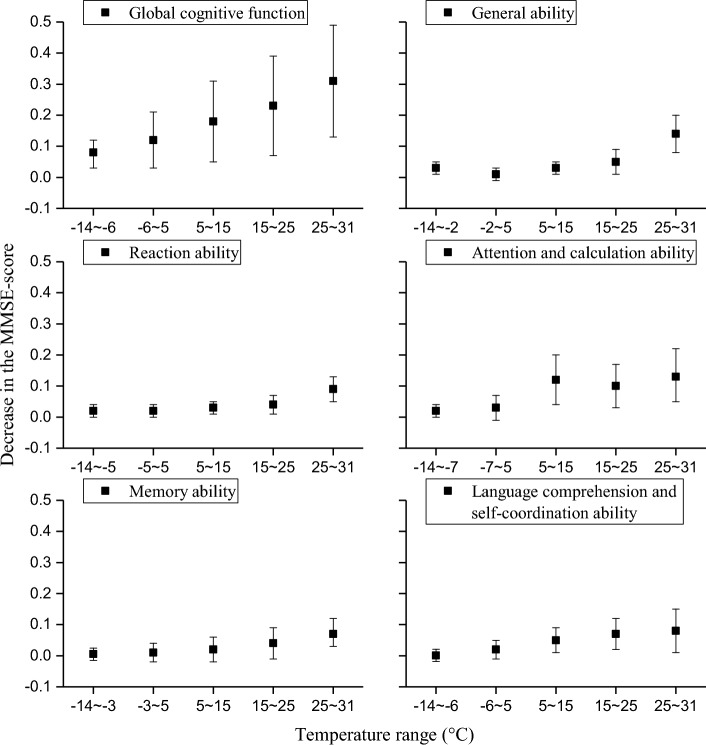
Decrease in the MMSE-score of various cognitive functions for every 1 °C rise (within the intervals of − 7 ~ 5 °C, 5 ~ 15 °C, and 15 ~ 25 °C) or drop (within the intervals of − 14 ~ − 6 °C, − 14 ~ − 2 °C and − 14 ~ − 7 °C) in monthly temperature.

Based on the relationship curve between temperature variation and the MMSE-score, we used the extended Bayesian spatiotemporal model to investigate the respective quantitative effect of temperature on the various cognitive functions of older adults (see Methods). The temperature range was divided into five intervals including − 14 ~ the reference temperature, the reference temperature ~ 5 °C, 5 ~ 15 °C, 15 ~ 25 °C, and 25 ~ 31 °C, in which the effect of every 1 °C rise or decrease of high and low temperature on MMSE-score was quantified. The temperatures of − 6 °C, − 2 °C, − 5 °C, − 3 °C, and − 6 °C accounted for the reference temperatures of the global cognitive function, general ability, reaction ability, attention and calculation ability, memory ability, and language comprehension and self-coordination ability of older adults, respectively (see Methods). Figure 4 exhibits that the effect of high temperature on the decline of cognitive function increases with the rise of temperature, in which the effect of high temperature culminates in the interval of 25–31 °C, and every 1 °C rise in monthly temperature corresponds to a decline of 0.32 (95% CI 0.14–0.51) in MMSE-score for the global cognitive function. The effect of high temperature on the general ability and memory ability of the sub-cognitive functions reached the maximum of 0.13 (95% CI 0.09–0.21) and the minimum of 0.08 (95% CI 0.04–0.13) in MMSE-score, respectively. Low temperatures were associated with the decline of global cognitive function, general ability, attention and calculation ability, whereas suggesting an insignificant or protective effect in terms of the reaction ability, memory ability, and language comprehension and self-coordination ability. The effect of low temperature on the global cognitive function, general ability, attention and calculation ability culminates in the intervals of − 14 ~  − 6 °C, − 14 ~ − 2 °C and − 14 ~ − 7 °C, corresponding to a decrease of 0.07 (95% CI 0.02–0.11), 0.04 (95% CI 0.02–0.06) and 0.03 (95% CI 0.01–0.05) in MMSE-score for every 1 °C drop in monthly temperature, respectively.

### Displacement effects of the low and high temperatures

**Figure 5:**
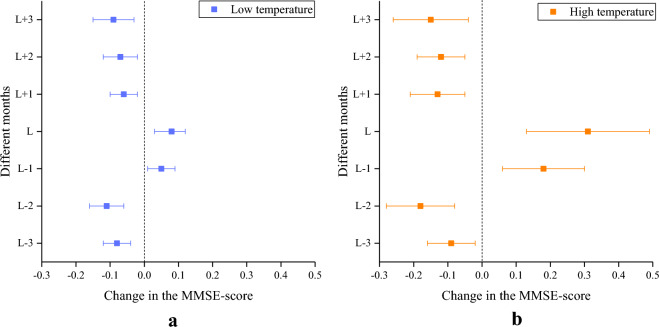
Displacement effects of low and high temperatures for the global cognitive function of older adults. (**a**) low temperature. (**b**) high temperature.

The displacement effects of temperature include two parts: the lag effect of temperature in the months before the current month and the lead effect of temperature in the months after the current month, both of which have been confirmed to exert a certain impact on various health outcomes^32,43^. Here, we used the improved Bayesian spatiotemporal model to assess the quantitative displacement effects of high temperature (i.e., temperature range of 25 ~ 31 °C) and low temperature (i.e., temperature range of − 14 °C ~ the reference temperature) on the cognitive function in older adults, including the global cognitive function and five sub-cognitive functions (see Methods). Figure 5 exhibited that for the displacement effects of low and high temperature on the global cognitive function, we did not find any displacement effect of monthly temperature except for the temperature of the current month (L) and the previous month (L-1). The displacement effects of temperature on the five sub-cognitive functions were shown in Supplementary Figs. 1–5, demonstrating the consistency with that of the global cognitive function, which indicated that both temperature in the current month (L) and the previous month (L-1) facilitated the decline of MMSE-score for the various sub-cognitive functions, and that the displacement effects of temperature in other months were insignificant or protective for the sub-cognitive functions. Thus in the subsequent analysis, we used the sum of the temperature effects of the current month and the previous month to reflect the overall effect of monthly temperature on cognitive function of older adults. Table 1 exhibits the overall effect of low and high temperature on the MMSE-scores of the global cognitive function and five sub-cognitive functions of older adults. The overall effect of low temperature on the decline of cognitive function is insignificant (the 95% confidence interval spans the value of 0) or protective except for the global cognitive function, general ability, and attention and calculation ability, whereas the overall effect of high temperature exerts a certain significant effect on the different categories of sub-cognitive functions, which is consistent with the results shown in Figs. 3 and 4.

**Table 1:** The overall effect of low and high temperature on the MMSE-scores of the global cognitive function and five sub-cognitive functions.

Categories of the cognitive functions	The overall effect of temperature and 95% confidence interval (CI)					
	Low temperature			High temperature		
	Calculated value	Lower limit of 95% CI	Upper limit of 95% CI	Calculated value	Lower limit of 95% CI	Upper limit of 95% CI
Global cognitive function	0.14	0.06	0.25	0.48	0.21	0.74
General ability	0.04	0.02	0.06	0.24	0.13	0.35
Reaction ability	0.03	0.01	0.05	0.16	0.08	0.24
Attention and calculation ability	0.03	0.01	0.06	0.22	0.08	0.34
memory ability	0.01	− 0.03	0.05	0.15	0.07	0.23
Language comprehension and self-coordination ability	0.01	− 0.01	0.03	0.14	0.03	0.22

### Heterogeneity across various socioeconomic and demographic characteristics

**Figure 6:**
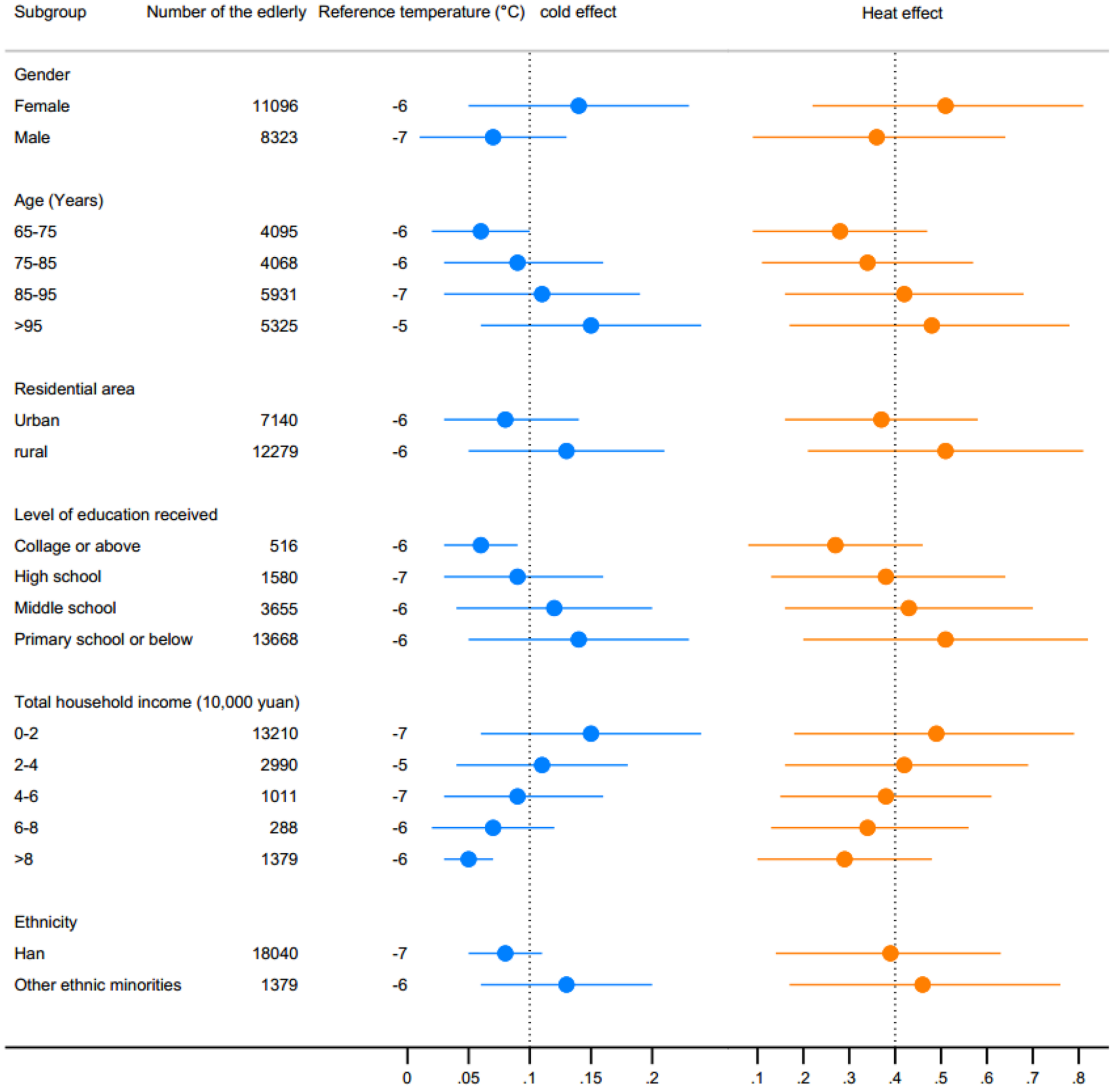
Heterogeneity for the MMSE-score of global cognitive function in older adults across various socioeconomic and demographic characteristics.

We explored the overall effect of monthly temperature on the global MMSE-score in multiple subgroups of older adults with respect to different socioeconomic and demographic characteristics, whose tolerance and adaptability to cold and heat differed upon exposure to low and high temperatures^15,24^. We assessed the significance of differences for the temperature effects across various subgroups (see Methods). Figure 6 illustrates the heterogeneity of the effects of low and high temperature on the decrease of the global MMSE-score of older adults in different subgroups, where the MMSE-score of the male older adult is less affected by temperature than that of the female subgroup, and older adults older than 95 years are more susceptible to the interference of external temperature relative to those of other age subgroups. The MMSE-score of older adults in rural areas exhibited a greater vulnerability to monthly temperature fluctuations compared with that in urban areas. Lower educational attainment of older adults was found to be associated with reduced cognitive function, and higher level of household income corresponded to weaker temperature effects on cognitive function compared with the counterpart subgroups. The impact of temperature on cognitive function of Han is less than that of other ethnic minorities. Overall, high temperature exposure exerts a greater negative impact on the MMSE-score of older adults than that of low temperature, which is consistent with the pattern of changes in the curve of monthly temperature and the global MMSE-score of older adults in Fig. 2.

### Sensitivity analysis

We explored the effect of temperature variation on the cognitive function for older adults using the Bayesian spatiotemporal model, controlling for several spatially and temporally structured and unstructured factors that may affect the results. To confirm the reliability of our results, based on model (3), the sensitivity analysis for various combinations of the interference factors in the Bayesian spatiotemporal model was conducted to estimate the overall effect of high and low temperatures on the MMSE-score of global cognitive function of older adults (see Methods). As shown in Supplementary Table 1, the results exhibit that the combination of various interference aspects in the model had less effect on the quantitative association between low or high temperature and the MMSE-score of global cognitive function, indicating the reliability of the study.

## Discussion

At present, global temperature sustains the rising trend, and the frequency of occurrence of extreme climate events involving extreme cold and heat weather is increasing, whose spread is expanding rapidly simultaneously^1,47^. Abnormal non-optimal temperatures account for the negative effects on multiple health outcomes ranging from mortality, respiratory disease, and suicide to reduced or impaired cognitive function due to high and low temperature exposure, which has been demonstrated in several researches^4,9,15,43^. Based on the dataset of CLHLS, our study identified and confirmed the detrimental effects of ambient low and high temperatures on cognitive function in older adults in light of the current climate background and the trends of temperature variation. To the best of our knowledge, this sample is currently the largest cognitive function examination dataset for older adults in China, and the sample size and information richness are superior to several previous studies related to temperature and cognitive function^15,22,48^.

The results showed that low and high temperatures are associated with lower examination scores of cognitive functions of older adults to varying degrees, which was mainly manifested in the effect of high temperature on the global cognitive function and five sub-cognitive functions. We found no significant association of low temperature on the decline of cognitive function except for the global cognitive function, general ability, and attention and calculation ability. Overall, the linkage between high temperature exposure and cognitive decline in older adults was demonstrated to be more prominent compared with that of low temperature. Several potential possible correlation-influence mechanisms account for these. Excessive heat exposure promotes the increased blood oxygen level-dependent activation of task-related regions dominated by the cerebral cortex, reducing hyperpyrexia-related neural efficiency. High temperatures elevate the plasma serotonin levels and suppress the productive capacity of neurotransmitter dopamine, which controls the performance of complex tasks ^49,50^. Heat-induced pathophysiological changes in the brain include enhancive vitality in the internal parietal sulci and limbic system, and reduced activity in the occipital, frontal, and temporal lobes, all of which are predisposing factors of decreased cognitive performance in older adults under high temperature^51^. Several physiological mechanisms exist to substantiate the decline in cognitive function correlated with low temperature simultaneously. Cold sensation causes vasoconstriction, reducing the capacity of blood circulation and oxygen supply in the brain, and induces a decrease in mitochondrial DNA copy number mediating the relationship between temperature and cognitive function^23^. Also, the retention of phosphorylated Tau is enhanced under cold condition simultaneously, which is the dominant factor in Alzheimer's disease^52^. Prior studies have confirmed the relationship between precipitation and cognitive function, where precipitation and cognitive function are negatively correlated^53,54^. Rain may discourage older adults from leaving their homes with potential consequences for social isolation, decreased physical activity, and cognitive decline^29,30^. Also, precipitation affects the concentrations of fine particulate matter (e.g., PM_2.5_) in the atmosphere^27,28^. The concentrations of particulate matter have been widely confirmed to be associated with cognitive decline^25,26^, whereby linking precipitation with cognitive decline. Therefore, we controlled for PM_2.5_ and precipitation as the interference factors in the model.

We found that older adults in the subgroups of rural area and low income acquired a lower cognitive function score than those of the urban and high-income subgroups in terms of low and high temperatures. This is because older adults households of low-income in rural areas use less heating in winter, and dwellings with fewer double/triple panes of glass than that of urban areas can better retain the heat loss of indoor^24^. The prevalence of air conditioning in rural areas in summer is much lower than that in high-income urban households, which is associated with a greater effect of high temperature on the cognitive function for the older adults in low-income households^55^. The majority ethnic Han was found to be less affected by temperature than that of the rest of the ethnic minorities, whose living environment and the construction facilities of household were easily interfered by external temperature^56^.

There are some limitations in this study. Our sample of cognitive function examination scores included a wealth of information with respect to the socioeconomic and demographic characteristics of older adults, and the sample size is superior compared to other researches^15,16,22^. However, due to the limited number of cases recruited, the statistical capacity of the model would be reduced, especially in terms of investigating the heterogeneity of differences in low and high temperature effects between various subgroups^16^. A larger sample covering more of the older adults would improve and address the assessment accuracy of the model. We did not involve the detailed usage of air conditioning in summer and precise data of heating in winter of the study, which overestimated the impacts of high and low temperature on cognitive function of older adults, respectively^15^. To avoid the aggregation effect of temperature grid size on temperature variations within cities, we used a high-resolution temperature grid to obtain the temperature exposure for older adults^34,36^. Higher resolution grids for temperature data could improve the accuracy of assessing temperature exposure in older adults. Due to the limitation of the temperature exposure range of the study subject, we were unable to quantify the effect of lower temperature exposure range and compare it with the effect of high temperature exposure range. More sufficient sample data would help understand the changing trends of cognitive function in older adults at lower temperatures.

The cognitive function status of older adults determines the social function and quality of life for older adults, which is related to the healthy and balanced development of society^16^. Our findings confirm that ambient high or low temperatures are associated with cognitive decline in Chinese older adults. The temperature interval of older adults involved in this study varied from − 14 to 31 °C, which is a broader temperature range compared to previous studies^15,16^. How cognitive function changes in older adults exposed to extreme heat (above 31 °C) or extreme cold (below − 14 °C) and the heterogeneity of cognitive function with respect to different socioeconomic demographic characteristics at these extreme temperature conditions need to be further investigated. Larger and broader samples of cognitive function would help identify and characterize the effects of extreme heat or cold on cognitive function in older adults in the future.

The results of the study contribute to and complement a complete presentation for fully understanding the relationship between environmental temperature and cognitive function in older adults, which contains critical reference consequence for identifying and assessing the influence of temperature on other health-related outcomes in the context of current climate change and temperature rise.

## Conclusion

The findings of the research suggest that monthly high temperature enhances both cognitive and sub-cognitive decline in older adults, whereas low temperature does not demonstrate a significant promoting effect except for the decline of global cognitive function, general ability, attention and calculation ability. Overall, the effect of high temperature on cognitive decline in older adults was significantly higher than that of low temperature. Our study provides key technical guidance for the formulation and embodiment of administrative intervention and precise health protection of cognitive function in specific external environment for the target-sensitive older population.

### Supplementary Information

Supplementary Information.

## Data Availability

The datasets generated and analyzed during the study are available from the corresponding author on reasonable request.
