# Supplementary material for: Ambient temperatures associated with reduced cognitive function in older adults in China
Source: Sci Rep. 2023 Oct 13;13:17414. doi: 10.1038/s41598-023-44776-2 (PMC10575877; doi:10.1038/s41598-023-44776-2)
Supplement: Supplementary file 1 — Supplementary Information. [file 41598_2023_44776_MOESM1_ESM.docx]

**Supplementary Material for**

**Ambient temperatures associated with reduced cognitive function in older adults in China**

Kun Hou^1*^, Xia Xu^2^

a b

Supplementary Fig. 1. Displacement effects of the low and high temperatures for the general ability of cognitive function. **a**, low temperature. **b**, high temperature.

a b

Supplementary Fig. 2. Displacement effects of the low and high temperatures for the reaction ability of cognitive function. a, low temperature. b, high temperature.

a b

Supplementary Fig. 3. Displacement effects of the low and high temperatures for the attention and calculation ability of cognitive function. **a**, low temperature. **b**, high temperature.

a b

Supplementary Fig. 4. Displacement effects of the low and high temperatures for the memory ability of cognitive function. **a**, low temperature. **b**, high temperature.

a b

Supplementary Fig. 5. Displacement effects of the low and high temperatures for the language comprehension and self-coordination ability of cognitive function. **a**, low temperature. **b**, high temperature.

Supplementary Table 1. The overall effect of high and low temperature on the MMSE-score of global cognitive function of the elderly with the control of different combinations of spatio-temporal interference factors, precipitation and PM_2.5_ in the model.

|  | The overall effect of temperature and 95% confidence interval (CI) | | | | | |
| --- | --- | --- | --- | --- | --- | --- |
| Confounder choice | Low temperature | | | High temperature | | |
|  | Calculated value | Lower bound of 95% CI | Upper bound of 95% CI | Calculated value | Lower bound of 95% CI | Upper bound of 95% CI |
| $\alpha_{\text{county }}+\beta_{\text{county }}\times\text{ time}$ | 0.14 | 0.06 | 0.24 | 0.46 | 0.18 | 0.76 |
| $\alpha_{\text{month }}+\beta_{\text{month }}\times\text{ time}$ | 0.12 | 0.05 | 0.23 | 0.44 | 0.19 | 0.75 |
| $\zeta_{\text{county-month }}+\psi_{\text{county-month }}\times\text{ time}$ | 0.14 | 0.06 | 0.26 | 0.45 | 0.17 | 0.74 |
| Precipitation | 0.13 | 0.04 | 0.24 | 0.46 | 0.18 | 0.75 |
| PM_2.5_ | 0.12 | 0.05 | 0.23 | 0.47 | 0.19 | 0.76 |
| $\alpha_{\text{county }}+\beta_{\text{county }}\times\text{ time}$, $\alpha_{\text{month }}+\beta_{\text{month }}\times\text{ time}$ 0.06 | 0.14 | 0.05 | 0.23 | 0.45 | 0.17 | 0.75 |
| $\alpha_{\text{county }}+\beta_{\text{county }}\times\text{ time}$, $\zeta_{\text{county-month }}+\psi_{\text{county-month }}\times\text{ time}$ | 0.12 | 0.04 | 0.23 | 0.46 | 0.18 | 0.74 |
| $\alpha_{\text{county }}+\beta_{\text{county }}\times\text{ time}$, precipitation | 0.13 | 0.05 | 0.24 | 0.45 | 0.17 | 0.76 |
| $\alpha_{\text{county }}+\beta_{\text{county }}\times\text{ time}$, PM_2.5_ | 0.14 | 0.06 | 0.22 | 0.44 | 0.16 | 0.75 |
| $\alpha_{\text{month }}+\beta_{\text{month }}\times\text{ time}$, $\zeta_{\text{county-month }}+\psi_{\text{county-month }}\times\text{ time}$ | 0.15 | 0.05 | 0.24 | 0.47 | 0.17 | 0.74 |
| $\alpha_{\text{month }}+\beta_{\text{month }}\times\text{ time}$, precipitation | 0.13 | 0.04 | 0.23 | 0.45 | 0.18 | 0.75 |
| $\alpha_{\text{month }}+\beta_{\text{month }}\times\text{ time}$, PM_2.5_ | 0.14 | 0.05 | 0.23 | 0.46 | 0.19 | 0.76 |
| $\zeta_{\text{county-month }}+\psi_{\text{county-month }}\times\text{ time}$, precipitation | 0.12 | 0.04 | 0.22 | 0.44 | 0.16 | 0.75 |
| $\zeta_{\text{county-month }}+\psi_{\text{county-month }}\times\text{ time}$, PM_2.5_ | 0.13 | 0.05 | 0.23 | 0.45 | 0.17 | 0.74 |
| Precipitation, PM_2.5_ | 0.14 | 0.06 | 0.24 | 0.45 | 0.19 | 0.75 |
